# The underperforming Abbott-Bioline Malaria Ag *P.f*/*P.v* rapid diagnostic test: a whiter shade of pale - where the truth is not plain to see

**DOI:** 10.1186/s12936-025-05638-6

**Published:** 2025-12-15

**Authors:** Kyaw Sithu Thein, Kyaw Soe, Saw Kan Sint Tun, Roy Kuipers, Sieb Janssen, Joel Tarning, Yinmon Htun, Nicholas P. Day, Thar Tun Kyaw, Arjen M. Dondorp, Nicholas J. White, Frank M. Smithuis

**Affiliations:** 1 Medical Action Myanmar, Yangon, Myanmar; 2 Mahidol-Oxford Tropical Medicine Research Unit (MORU), Faculty of Tropical Medicine, Mahidol University, Bangkok, PO 10400 Thailand; 3 Centre for Tropical Medicine and Global Health, Nuffield Department of Medicine, University of Oxford, Oxford, OX3 7BN UK

**Keywords:** Malaria, Rapid diagnostic tests, Abbott-Bioline™ Malaria Ag *P.f*/*P.v*, *P. falciparum*, *P. vivax*, Parasite density, ImageJ^®^

## Abstract

The Abbott-Bioline™ malaria antigen *Plasmodium falciparum* and *Plasmodium vivax* rapid diagnostic test (Abbott-Bioline™ Malaria Ag *P.f*/*P.v* RDT) is used widely in the Greater Mekong Subregion. It is approved by the World Health Organization (WHO) and deployed on a large scale in the regional malaria elimination programmes. Following reports of false-negative test results from field-workers, the Abbott-Bioline™ Malaria Ag *P.f*/*P.v* RDT was compared with microscopy in 187 persons with fever during a vivax malaria outbreak in Myanmar. Among them 11 tested positive for *P. vivax* using the Abbott RDT, while 46 were diagnosed with *P. vivax* malaria by microscopy. The sensitivity of the Abbott RDTs among febrile patients was 0.24 (95% CI 0.12–0.36) with a specificity of 1.0. Twenty-eight (61%) of the 46 patients had a parasite count of ≥200/µL, the WHO-recommended lower limit of detection for RDTs. Among these 11 were detected by the Abbott malaria RDT, a sensitivity at this threshold of 0.39, (95% CI 0.21–0.57). The colour band denoting positivity on the malaria RDT was often absent. The low intensity of the RDT colour band was confirmed objectively using ImageJ^®^, an image processing software. The Abbott-Bioline™ Malaria Ag *P.f*/*P.v* RDT is insensitive compared to microscopy. In remote areas where there is very limited access to health care and usually only a single source of diagnosis and treatment, as in rural Myanmar, this has resulted in dangerous delays in patients receiving appropriate treatment. The WHO Incidents and Substandard/Falsified Medical Products Team was informed of the problem, but did not respond effectively. Deployment of the underperforming malaria RDT continues.

**Supplementary Information:**

The online version contains supplementary material available at 10.1186/s12936-025-05638-6.

## Introduction

In response to the threat of worsening artemisinin and partner drug resistance in *Plasmodium falciparum*, the countries of the Greater Mekong Subregion (GMS) in Southeast Asia agreed to aim for elimination of falciparum malaria by 2025 [1, 2]. To coordinate the elimination effort, the Regional Artemisinin-resistance Initiative was established in 2014, with generous funding provided by the Global Fund, along with other international partners and national programmes.

Malaria in the region is prevalent predominantly in remote and difficult-to-access communities. This is especially true in Myanmar, which accounts for the large majority of all the reported malaria cases in the region [2–4]. In previous times, the diagnosis of malaria was made by a well-trained laboratory technician who stained a blood film and used a microscope to identify parasite densities of approximately 50 parasites/µL or higher. However, the trained staff, good microscopes and support for reagents were typically not available in remote communities. For microscopy-based diagnosis and treatment, people had to travel to distant clinics and hospitals. Transportation was both difficult and expensive [4, 5]. This situation changed dramatically after the introduction of community health workers (CHWs) trained and equipped with malaria rapid diagnostic tests (RDTs) who provided point-of-care diagnosis and effective treatment of confirmed malaria cases. The RDTs detect *P. falciparum* histidine-rich protein 2 (HRP2) for the diagnosis of falciparum malaria and *Plasmodium* lactate-dehydrogenase (pLDH) or aldolase for both *P. falciparum* and the other human malaria parasites in patients. These tests generally have diagnostic sensitivity close to that of good microscopy. The current World Health Organization (WHO) defined minimum lower level of detection for malaria RDTs is a parasite density of 200 parasites/µL [6]. The RDTs are simple to perform and interpret by the CHWs with minimal training and they provide actionable results within 15–20 min. Villagers in endemic areas have greatly appreciated the services provided by the CHWs which allow definitive malaria treatment to be given close to home. The uptake of malaria RDTs in remote rural Myanmar has been very high. The widespread coverage of CHWs, using sensitive RDTs and providing effective antimalarial treatment, also substantially reduced the time patients were infectious and, therefore, rapidly reduced malaria transmission in large part of the GMS. In many communities, malaria was eliminated as a consequence [4, 5].

## Historical background

In the past few years, there have been concerning reports in the region over the sensitivity of the Abbott malaria RDTs to diagnose clinical malaria. The Abbott-Bioline™ Malaria Ag *P.f*/*P.v* RDT is the most commonly used brand in Vietnam, Cambodia, Laos, and Myanmar. In 2022 and 2023, in an extremely remote area of western Myanmar, CHWs supported by the Global Fund and working for Medical Action Myanmar (MAM) reported concerns about the performance of the Abbott-Bioline Malaria Ag *P.f*/*P.v* RDTs. These CHWs, who had many years of experience using RDTs, observed that the tests were “not functioning as well as before” and suspected an increase in false-negative results. They reported many cases where patients with fever initially tested negative, only to test positive several days later as symptoms persisted or worsened. Five young children with fever reportedly died from *P. falciparum* malaria after repeatedly testing negative with these RDTs in the days leading up to their eventual positive results. A 25-year old medical assistant, employed by MAM in the same region, developed a fever and tested negative with the Abbott-Bioline Malaria Ag *P.f*/*P.v* RDTs for four consecutive days. On the fifth day, in addition to continued high fever, he had epistaxis and melaena and was referred by boat to the regional hospital. On the boat, he finally tested positive for falciparum malaria with the 5th Abbott RDT. In the regional hospital, he was diagnosed with severe falciparum malaria and treated accordingly (Medical Action Myanmar. *Progress report to UNOPS*. [Unpublished reports] Yangon: 2022 and 2023). These alarming reports clearly required further investigation. MAM therefore sent a medical team with microscopists to the area to compare the performance of the RDTs with microscopy in 2023. Unfortunately, the team was unable to reach the affected communities because of ongoing armed conflict in the region.

In June 2024, CHWs working for the Shoklo Malaria Research Unit (SMRU) and the Borderland Health Foundation, organizations based on the northwestern Thailand-Myanmar border which provide support for malaria control activities in the East of Myanmar, also reported false-negative results with the same Abbott malaria RDTs. SMRU subsequently conducted a prospective investigation to assess the quality of the Abbott-Bioline Malaria Ag *P.f*/*P.v* RDTs, comparing microscopy, the Abbott RDT and a different brand of RDT. In August 2024, they presented their report to the WHO Incidents and Substandard/Falsified Medical Products Team (WHO-ISF) and to Abbott Diagnostics Korea Inc. This report strongly suggested that the Abbott-Bioline Malaria Ag *P.f*/*P.v* RDTs failed to diagnose the majority of both falciparum and vivax malaria cases that were identified both by microscopy and by another RDT (First Response^®^). This detailed study and other anecdotal reports from the region raised serious concerns about the reliability of the Abbott-Bioline Malaria Ag *P.f*/*P.v* RDTs in routine use [7, 8].

The manufacturer, Abbott, responded, but did not accept that the RDTs underperformed when stored and used properly, although there had been no changes in the way these tests had been used and stored, and they had worked well previously, and the comparator tests were stored and used under the same conditions. The WHO-ISF team was also informed of the concerns, but neither Abbott nor the WHO took visible public action to inform medical professionals or the public about the potential risks associated with false-negative results from these specific RDTs. As a result, Abbott-Bioline Malaria Ag *P.f*/*P.v* RDTs continued to be used widely and on a large scale, as concerns about their reliability mounted.

MAM has been introducing CHWs to provide early diagnosis and treatment for malaria in remote areas of Myanmar since 2011 and is currently supporting a network of over 2000 CHWs. In December 2024, MAM was informed about a malaria outbreak in a remote area in Northwest Myanmar and, as part of the containment response, conducted an outbreak investigation with active case detection. As the reported lack of sensitivity of the Abbott-Bioline Malaria Ag *P.f*/*P.v* RDTs was an ongoing concern, and this was the only RDT provided by the donor UNOPS (United Nations Office for Project Services) and approved by the authorities, all patients presenting with fever had blood tested using both the Abbott-Bioline Malaria Ag *P.f*/*P.v* RDTs and microscopy, to ensure diagnostic accuracy. The results are reported below.

## Methods

### Malaria diagnosis

The malaria outbreak investigation was conducted in December 2024 in Sin Thay village, Hkamti district, in Sagaing division in Northwest Myanmar, approximately 50 km from the Indian border. Hkamti lies on the border between a tropical and a temperate climate zone, with a dry winter and a rainy monsoon season from May to October. Average monthly high temperatures range from 25 to 33 °C, while lows vary between 11 and 25 °C.

### Malaria RDT

**Fig. 1:**
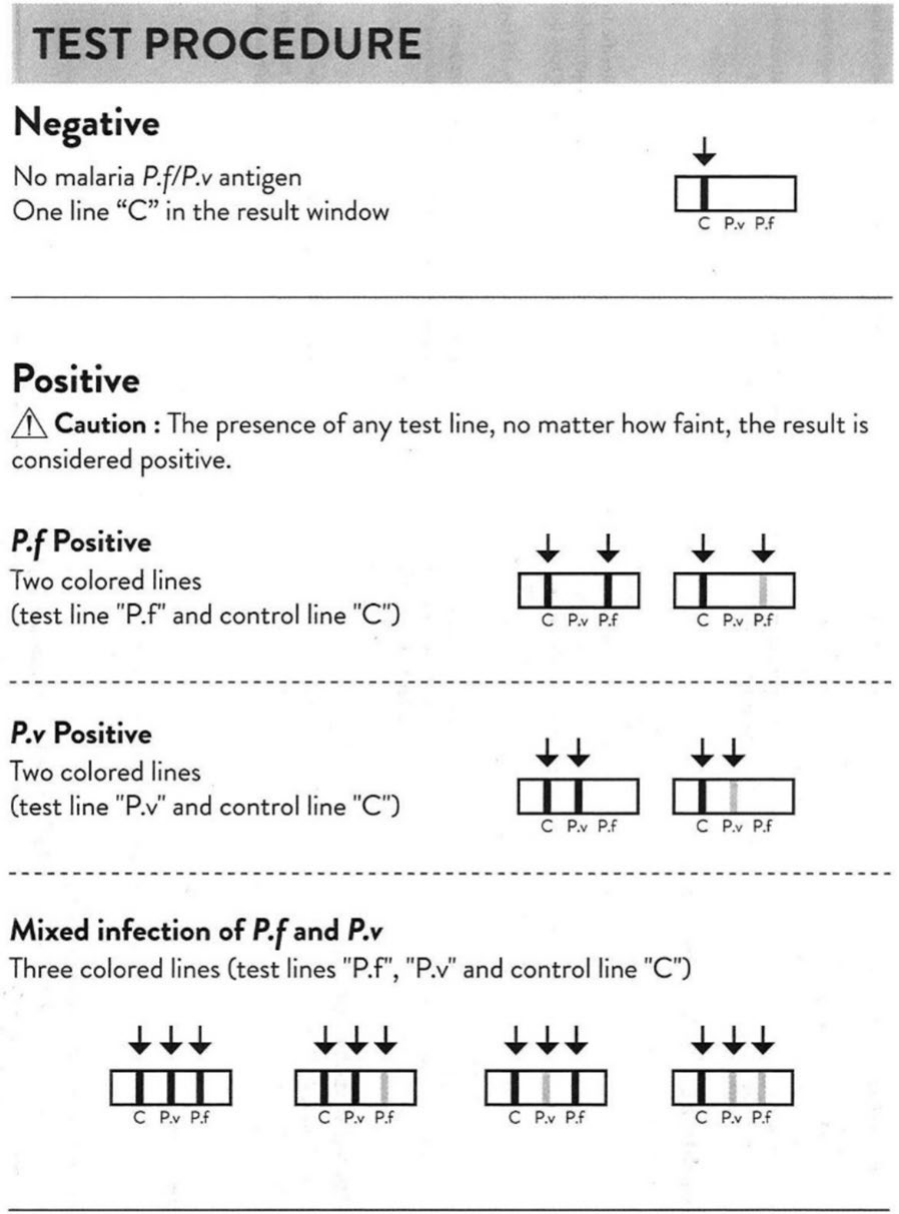
The test procedure instruction on the Abbott RDT instruction leaflet, routinely accompanying the product

The Abbott-Bioline™ Malaria Ag *P.f*/*P.v* RDT, manufactured by Abbott Diagnostic Korea, is a rapid, qualitative diagnostic test used to detect malaria antigens HRP2 and pLDH. In the outbreak investigation, RDTs were used by experienced health workers. All villagers in the outbreak area were invited for malaria screening. This was done with the Abbott-Bioline™ Malaria Ag *P.f*/*P.v*, batch 05DDI027A (product code—05FK86, expiry date 10-Sep-2025) under usual field conditions. Finger-prick blood samples were collected using the manufacturer’s provided alcohol swabs, lancets and specimen collection inverted cups to collect 5 μL of blood, which was then dispensed into the specimen well of the RDT. Four drops of assay diluent were dispensed in the diluent well and then the test was read after 15–20 min as per manufacturer instructions. The test result can be interpreted as negative, *P. falciparum*, *P. vivax* or mixed infection (both *P. falciparum* and *P. vivax*). All RDTs were inspected visually by two experienced health workers, independently from each other. The RDT test line colour (visual) intensity was scored as follows;

- *Negative*; no line colour was seen by both health workers.
- *Positive*; clearly visible, i.e. the intensity of the line colour was equal to or stronger than the faint line on the standard Abbott RDT instruction leaflet accompanying the product (Fig. 1).
- *Faint*; visible, but the intensity of the line colour was lower than that of the faint line on the standard Abbott RDT instruction leaflet accompanying the product.
- *Very faint*; only barely visible by 1 of the 2 health workers.

All positive, faint and very faint lines were considered RDT positive. In case of disagreement, specifically between a “negative” and an “very faint” line result, the RDT was classified as positive. When the control line (C) did not appear, the test was called “invalid” (although this did not happen). After reading the RDT result, a photograph was taken of the RDT. These photographs were later also used to measure the density of the RDT signal with the ImageJ^®^ tool.

### Microscopy

All individuals who complained of fever were invited for testing by microscopy. In addition, one individual who did not have symptoms, but tested positive with the RDT, was also tested by microscopy, although this was outside the routine practice.

For microscopy standard thick and thin blood films were prepared. The slides were air-dried, and stained with Giemsa’s stain (pH 7.2) for 15 min. Slides were read independently by two experienced microscopists using a light microscope. Slides were examined for up to 200 high power microscope fields before a slide was considered to be negative. If the slide was positive, parasite density was counted relative to leukocytes in the thick blood film, assuming a standard whole blood leukocyte count of 8000/μL. Parasites were routinely counted up to 200 leukocytes and in case parasites were not yet detected, the search was extended until 500 leukocytes were counted. All individuals who tested positive with either test were given antimalarial treatment according to the National Malaria Control Programme (NMCP) guidelines.

### Colour intensity assessment

**Fig. 2:**
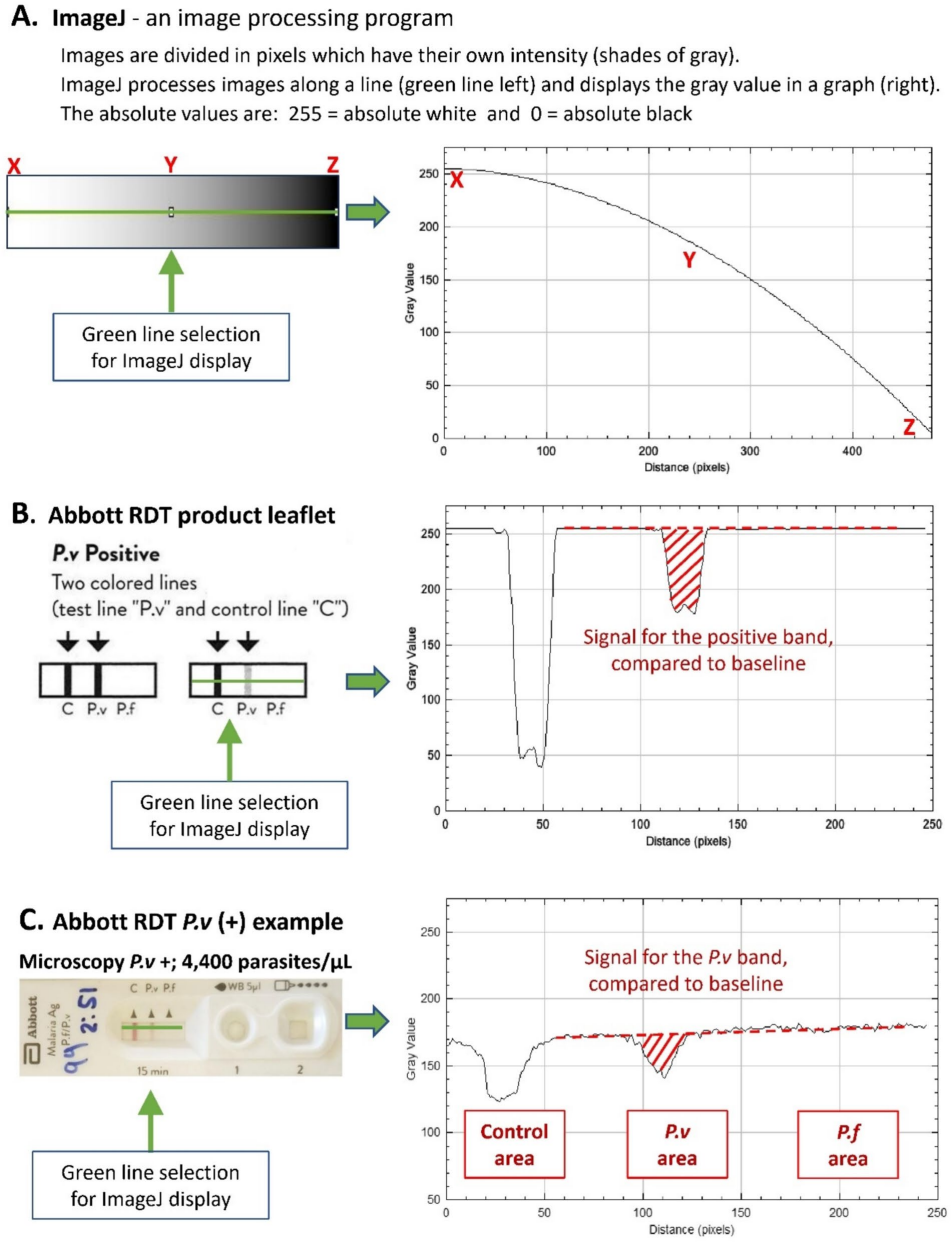
Image J profile examples. **A.** A selection of the rectangular shape (the green line in the left image) processed with ImageJ and displayed the grey density profile in the graph on the right. **B.** A selection from the Abbott RDT product leaflet, showing the example of the faint *P.v* line. **C.** A selection of the results window area of Abbott RDT from a patient who was *P.v* (+) with a parasite count of 4400/µL. Note; the white area of the result window is not absolute white (value; 255), but in this slide approximately 170–180, because of some colouring of the flow area by blood pigment

To measure the strength of the colour signal on the RDT objectively, we used ImageJ (version 1.53)^®^, a Java-based image processing program developed at the National Institutes of Health and the Laboratory for Optical and Computational Instrumentation (University of Wisconsin). ImageJ can analyse 8-bit colour and grayscale point images. It can read many image file formats, including JPEG as well as raw formats [9]. ImageJ processes images along a user defined selection and display a grey density line profile in a graph. The absolute grey value scores are 255 for absolute white and 0 for absolute black (Fig. 2).

All RDTs from patients who tested positive by microscopy with a parasitaemia of ≥200/µL were scanned using ImageJ and the corresponding density line profiles were presented in graphs. We routinely selected the centre of the RDT results window for analysis, but when a faint test line appeared non-homogeneous, an additional area showing the strongest visible colour was also selected. For each RDT, the pixel density profile with the highest intensity was used. The signal of the *P.f* band and the *P.v* band were compared to the baseline signal (i.e. the white area after processing the test) over the result window. It should be noted that the baseline signal varies as the result window is typically discoloured by blood pigment. As a result, the baseline is not 255 (pure white) but a variably lower value. A positive signal was defined as clearly distinguishable from the baseline. A weak signal was defined as a difference in grey value score from the baseline of 9 or less.

### Data analysis

The diagnostic performance of the RDT on symptomatic individuals was expressed in terms of sensitivity, specificity, positive predictive value (PPV), and negative predictive value (NPV), using microscopy results as the gold standard. Patients with microscopy results indicating *P. falciparum* gametocytes only were classified separately.

### Consent to participate

This is a retrospective analysis of routine observations and additional informed consent was not required.

## Results

### RDT and microscopy results

On 3, 4 and 5 December 2024, active case detection was conducted in Sin Thay village in response to the reported malaria outbreak. All residents were invited for malaria testing and a total of 914 individuals were tested with the Abbott-Bioline Malaria Ag *P.f*/*P.v* RDT.

**Fig. 3:**
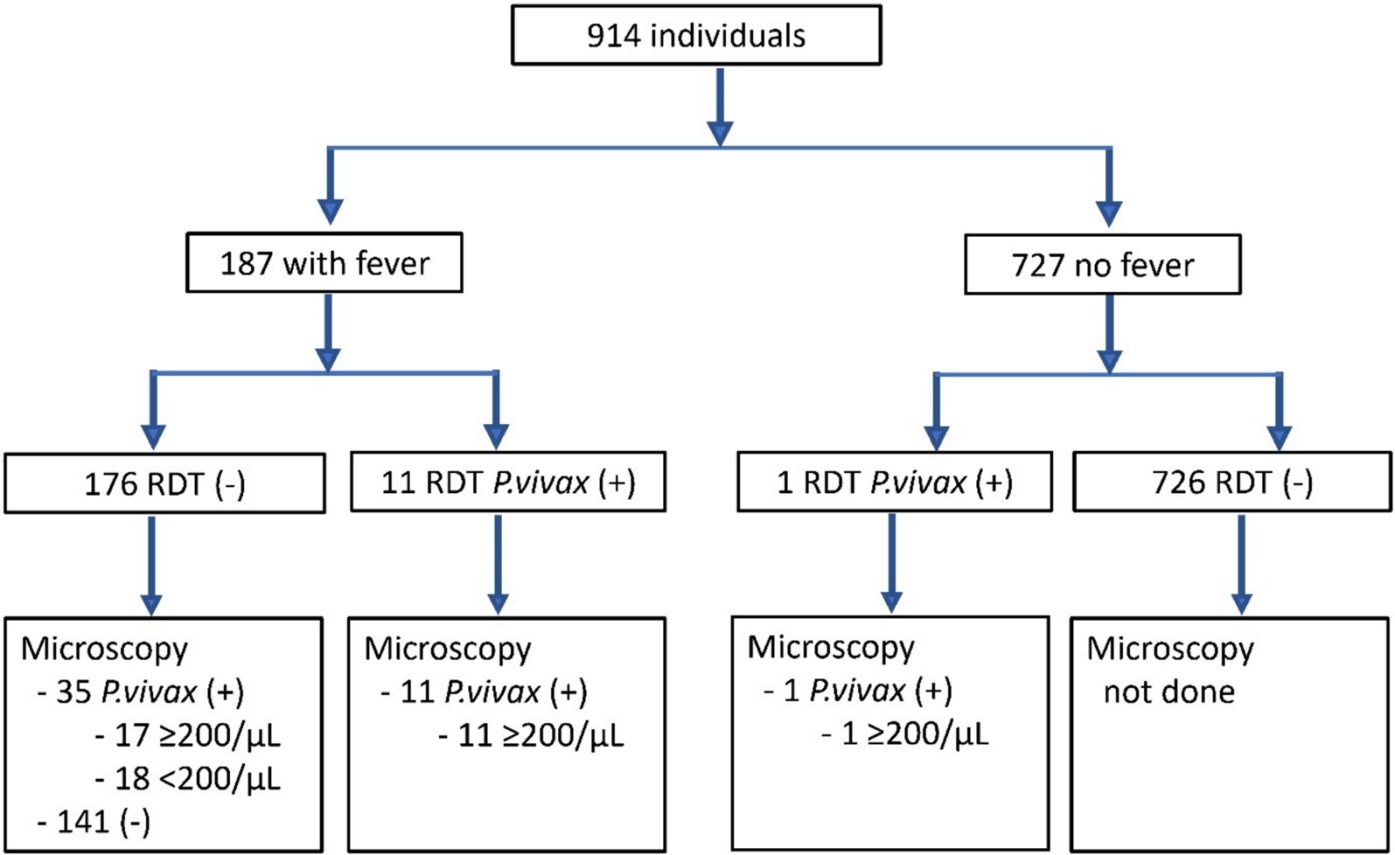
Malaria survey results

Among the villagers, 187 persons complained of fever and blood samples were taken for microscopy. Of these 187, 11 tested positive for *P. vivax* with the Abbott RDT while microscopy identified 46 with *P. vivax* malaria and 1 with *P. falciparum* (gametocytes only). A parasite count of ≥200/µL, the WHO-recommended minimum parasite density detectable by RDT, was observed in 28 (61%) of the 46 persons with vivax malaria. Malaria survey results are shown in Fig. 3.

In the quality control assessment of microscopy there was 100% agreement in the identification of malaria species between the two microscopists. The mean difference in the log parasite counts recorded by the two checking microscopists was 6.2% (95% CI 0.1–12.3%).

In summary, in this malaria outbreak survey the sensitivity of the Abbott RDTs among the 187 persons with fever was 0.24 (95% CI 0.12–0.36) with a specificity of 1.0. The PPV and NPV were 100 and 80.1% (95% CI 74.2–86.0), respectively. Among individuals with a patent parasitaemia of ≥200 parasites/µL, the sensitivity of the Abbott RDT was 0.39, (95% CI 0.21–0.57) with a specificity of 1.0. The PPV and NPV in this group were 100% and 90.3% (95% CI 85.9–94.6), respectively.

Among the 727 asymptomatic individuals, one person also tested positive for *P. vivax* with the Abbott RDT. Although this was not part of routine practice, this individual was also checked with microscopy and was found to be positive for microscopy with a parasitaemia of ≥200 parasites/µL.

### Strength of the signals on the RDT judged by eye and ImageJ^®^

**Fig. 4:**
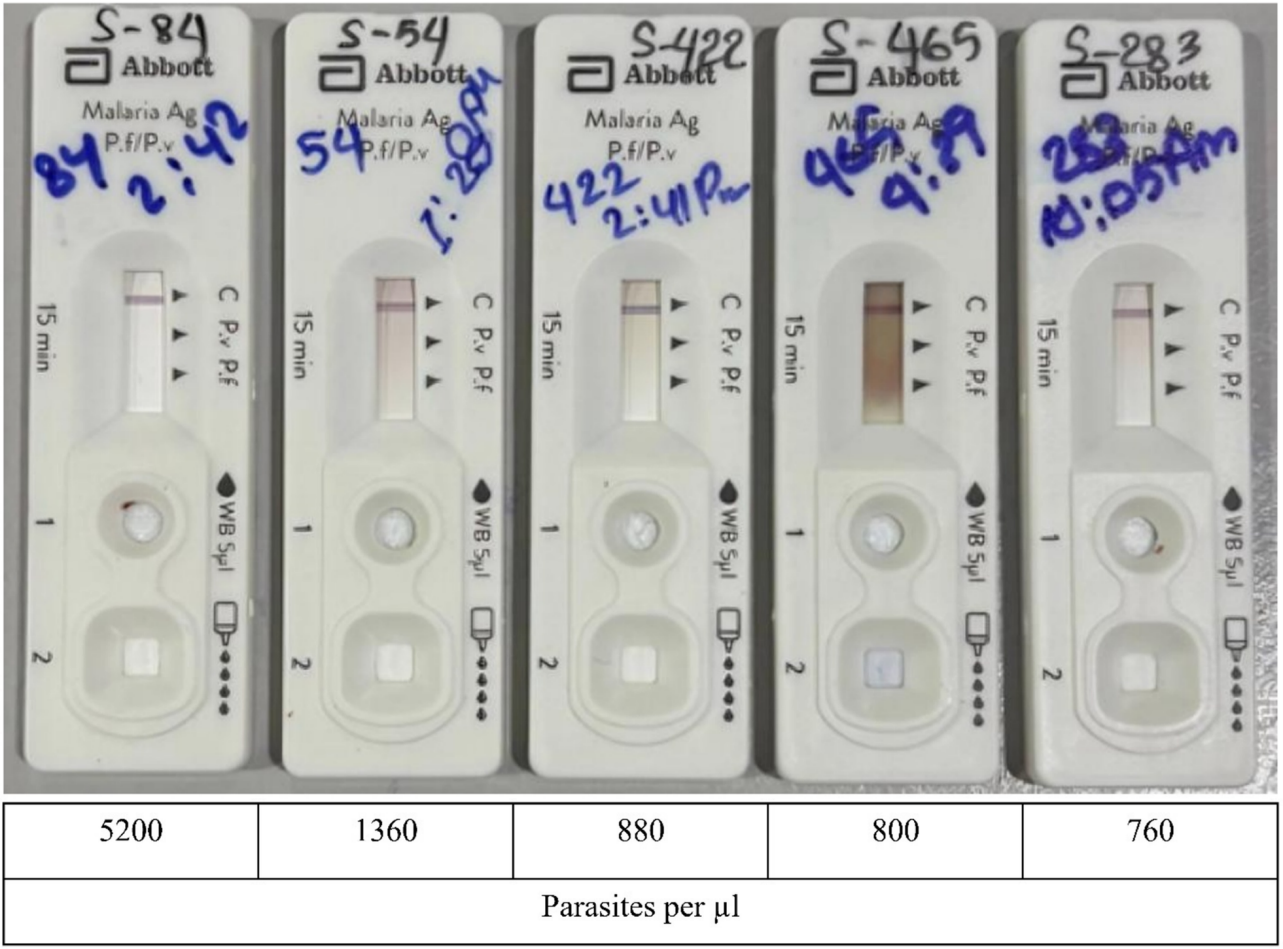
Photographs showing five RDTs from microscopy-positive patients with *P. vivax* parasite counts ≥200/µL (the WHO-recommended minimum density detectable by RDTs) which were classified as “negative” by the health staff, presented with their associated microscopy parasite counts

The two staff who read the RDTs identified 12 tests as positive for *P. vivax*. Of these, 10 were classified as “faint” and 2 as “very faint”. Examples of the 17 RDTs that were considered “negative” with patent parasitaemia of ≥200 parasites/µL detected by microscopy are shown together with their associated parasite counts in Fig. 4.

All RDTs from patients who tested positive by microscopy with parasitaemia ≥200/µL were scanned and analysed using ImageJ. The analysis produced by ImageJ was in line with the visual judgement of the 2 staff. While faint lines produced a clear signal in the ImageJ graphs, very faint RDT lines had a very weak signal, and all RDTs that were false negatives produced no detectable signal with ImageJ (Table 1; Supplementary Fig. S1).

**Table 1:** Parasite counts of microscopy positive patients, compared to the Abbott-Bioline Malaria Ag *P.f*/*P.v* RDT results, the ImageJ signal of the *P.v* band and the estimated grey value deviation from baseline

No	Age	Sex	*P. vivax* parasites (/µL)	RDT visual intensity rating	RDT ImageJ signal rating	Grey value deviation from baseline	RDT recorded
1	5	Male	9680	Faint	+	>20	*P.v*
2	8	Male	6960	Faint	+	10–20	*P.v*
3	8	Male	5200	Not visible	No signal	–	False neg
4	6	Male	4400	Faint	+	>30	*P.v*
5	7	Male	4320	Faint	+	10–20	*P.v*
6	7	Male	3040	Faint	+	10–20	*P.v*
7	10	Male	2960	Faint	+	10	*P.v*
8	5	Male	2880	Faint	+	10–20	*P.v*
9	18	Female	2520	Faint	+	10–20	*P.v*
10	9	Male	1800	Very faint	Weak signal	<10	*P.v*
11	14	Male	1360	Not visible	No signal	–	False neg
12	8	Male	1000	Faint	+	>30	*P.v*
13	17	Male	880	Not visible	No signal	–	False neg
14	1	Female	800	Very faint^a^	Weak signal	<10	*P.v*
15	10	Male	800	Not visible	No signal	–	False neg
16	8	Male	760	Not visible	No signal	–	False neg
17	16	Female	560	Faint	+	10–20	*P.v*
18	10	Male	360	Not visible	No signal	–	False neg
19	17	Male	360	Not visible	No signal	–	False neg
20	12	Female	320	Not visible	No signal	–	False neg
21	11	Female	280	Not visible	No signal	–	False neg
22	14	Female	240	Not visible	No signal	–	False neg
23	16	Male	240	Not visible	No signal	–	False neg
24	9	Male	240	Not visible	No signal	–	False neg
25	17	Male	240	Not visible	No signal	–	False neg
26	12	Female	200	Not visible	No signal	–	False neg
27	17	Male	200	Not visible	No signal	–	False neg
28	14	Female	200	Not visible	No signal	–	False neg
29	19	Female	200	Not visible	No signal	–	False neg
30	6	Female	160	Not visible	N/A		False neg
31	9	Male	160	Not visible	N/A		False neg
32	9	Male	160	Not visible	N/A		False neg
33	11	Male	120	Not visible	N/A		False neg
34	11	Male	120	Not visible	N/A		False neg
35	12	Male	80	Not visible	N/A		False neg
36	13	Male	80	Not visible	N/A		False neg
37	13	Male	80	Not visible	N/A		False neg
38	11	Male	40	Not visible	N/A		False neg
39	11	Female	40	Not visible	N/A		False neg
40	3	Female	40	Not visible	N/A		False neg
41	18	Female	40	Not visible	N/A		False neg
42	16	Female	32	Not visible	N/A		False neg
43	15	Male	16	Not visible	N/A		False neg
44	11	Male	16	Not visible	N/A		False neg
45	13	Male	16	Not visible	N/A		False neg
46	19	Female	16	Not visible	N/A		False neg
47	16	Female	16	Not visible	N/A		False neg

^a^Scored as “not visible” by 1 staff and “very faint” by the other staff

**Fig. 5:**
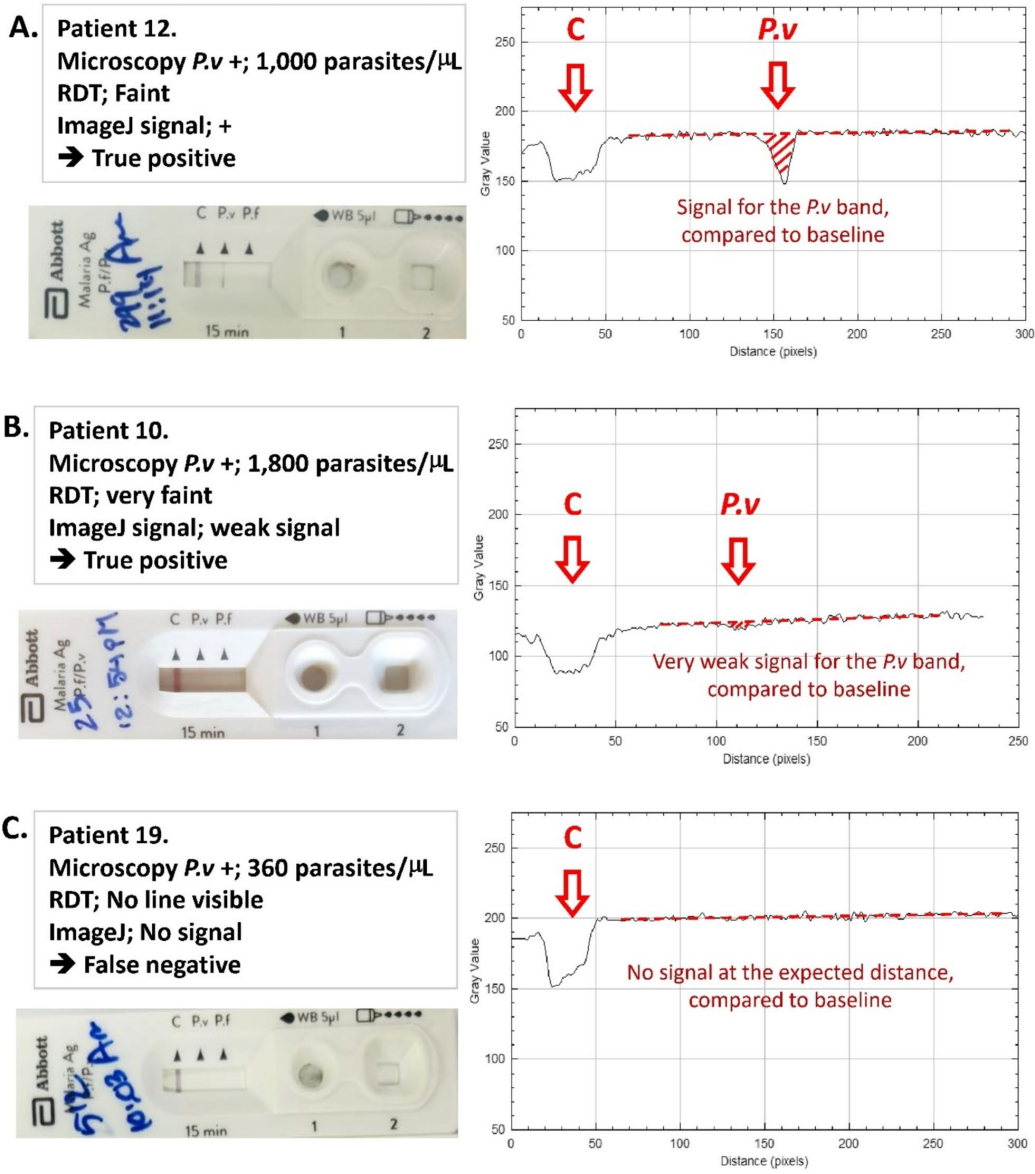
Examples of RDTs from 3 patients who tested positive by microscopy with parasite counts ≥200/µL, considered by the health staff a having **A.** a faint line, **B.** a very faint line and **C.** no visible line, together with their corresponding ImageJ grey density line profile graphs and the associated parasite count

Examples of RDTs with a faint line, a very faint line and a false negative RDT with their associated ImageJ signals and the associated parasite counts are presented in Fig. 5. The complete set of RDT images from 29 patients who tested positive by microscopy with parasite counts ≥200/µL, along with their corresponding ImageJ grey density line profile graphs, is presented in supplementary Figure S1.

## Discussion

Malaria RDTs are an essential component of routine diagnosis and treatment. They are particularly valuable in remote areas where other diagnostic methods are not available. They are invaluable in outbreak investigations. In the GMS there have been recent concerns about the sensitivity of the widely used Abbott-Bioline Malaria Ag *P.f*/*P.v* RDTs. During this investigation, conducted in a malaria outbreak response in Northwest Myanmar, the Abbott-Bioline Malaria Ag *P.f*/*P.v* RDTs had a sensitivity of only 24% compared to microscopy for detecting *P. vivax* malaria. In patients with a parasite density ≥200/µL, which is the WHO-recommended minimum parasite density that should be detectable by RDT, the sensitivity was 39%. These results are clearly unacceptable.

Only a small number of RDTs was evaluated from one batch of Abbott-Bioline Malaria Ag *P.f*/*P.v* RDTs, but these finding add credence to the anecdotal reports received from our own teams. They are also in line with other findings in the region, in particular the detailed investigations conducted on the Thailand-Myanmar border which cross-checked a much larger number of RDTs, of various lot numbers, and compared them with microscopy and another manufacturer’s RDTs [7]. In addition, there have been anecdotal concerning reports from the NMCP in Vietnam in 2023 (Regional Artemisinin-resistance Initiative (RAI) Independent Monitoring Panel visit to Lai Chau Province, Viet Nam, 12–19 May 2023. [Unpublished report]) and during the Myanmar VBDC/NMCP annual meeting in March 2025. All these findings point in the same direction; the current Abbott-Bioline Malaria Ag *P.f*/*P.v* RDTs have low diagnostic sensitivity.

This appears to be a new problem. Abbott-Bioline Malaria Ag *P.f*/*P.v* RDTs and their predecessors have been used satisfactorily by the more than 2000 project CHWs in Myanmar for over 10 years without any major complaints until 2022. The reason for the recent decline in performance remains unclear.

Incorrect storage at high temperatures, above 40 °C, cannot be ruled out, although this has not changed substantially in the past decade. If incorrect storage were to explain this poor performance then, as the maximum temperature in Northwest Myanmar where the RDTs were used, is below 40 °C, any potential storage issues must have occurred before the RDTs arrived at the project site. This is possible but unlikely, as the transport and storage methods have remained consistent over time, do not appear to affect other manufacturer’s RDTs, and performance issues with Abbott RDTs stored under the same conditions had not been reported previously. Moreover, the effects of transportation and storage were assessed by the SMRU investigations and ruled out as factors explaining their poor test results [7].

A potential explanation for the poor test results, suggested by both Abbott and WHO, was the poor visual acuity of the testers, i.e. that the health staff may have misread RDT results, failed to detect faint test lines, and interpreted them as negative. The Abbott RDT instruction leaflet states; “The presence of any test line, no matter how faint, the result is considered positive”. The phrase “no matter how faint” is ambiguous as it does not provide a clear or practical cut-off point. Visual acuity naturally varies among health workers but RDTs should not be designed to give faint lines which could easily be missed. In this particular case, even objective image analysis with a well-known image processing program, could not detect a large proportion of tests which should have been positive. If the test line indicating a positive malaria result is too faint to be detected by the human eye (or an image processing program), despite the presence of patent parasitaemia, then the RDT cannot be considered fit for purpose.

**Fig. 6:**
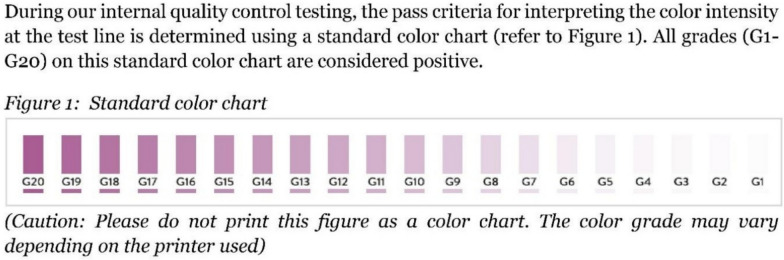
The new “standard colour chart” sent in a letter (date 17 November 2024) from Abbott Diagnostics Korea

On 17 November 2024, Abbott sent a letter to SMRU together with a new “standard colour chart” that had not been shared previously and, to date, has still not been shared with other customers (Abbott Diagnostics Korea Inc. Regarding reported false negatives and faint test lines [Unpublished letter]. Sent to: SMRU. 15 November 2024.). This chart (Fig. 6) is accompanied by a caution advising that it should not be printed as “the colour grade may vary depending on the printer used”. The faintest colour on the chart (G1) has a value of 2 on a scale from 0 to 255 when measured using ImageJ. This level of faintness falls well within the range of discoloration caused by contamination from blood and other substances on the RDT result window, making it almost certain to be missed. This seems to accept low test sensitivity.

The adverse impact of the large-scale use of low-sensitivity malaria RDTs is difficult to estimate but is likely to be serious. A negative malaria RDT result should not be considered definitive in a patient with persistent or deteriorating symptoms in the absence of another explanation for the symptoms. Referral to hospital for such cases is recommended. However, malaria is often concentrated in the most remote areas with very poor infrastructure and no public transport. Referral to a higher level with microscopy generally takes many hours—often days—and is prohibitively expensive. This underlines the importance of good quality malaria RDTs. Delayed and missed diagnoses of malaria increase the risk of progression to severe complications and death. In areas that MAM serves five young children reportedly died from falciparum malaria after repeatedly testing negative with the Abbott-Bioline Malaria Ag *P.f*/*P.v* RDTs in the days preceding their eventual positive results. It was only because the parents kept returning to the community health workers for retesting their children, that we were able to confirm malaria as the cause of death. Most patients are unlikely to have been as persistent, and would have accepted that they did not have malaria. In the remote areas where malaria is prevalent untreated falciparum malaria may well result in death. Such deaths are likely either to not be registered or not be reported as malaria-related deaths (as malaria had been “excluded”). This results in severe underreporting. Beyond the immediate risk of missed diagnoses, the widespread use of low-sensitivity malaria RDTs extends the time between the patients becoming infectious and receiving treatment. As a result, the duration of infectiousness is prolonged, leading to increased malaria transmission [10].

Whereas there is a clear pathway for the assessment and response to low quality medicines, this seems less clear for diagnostics. These concerning findings were reported to both the WHO-ISF team and Abbott, the manufacturer. Earlier, SMRU had reported their findings in August, September, October and November 2024. Neither the WHO-ISF or Abbott accepted that there was a serious problem initially and so no visible public action was taken to inform medical professionals or the public about the potential risks associated with false-negative results from these RDTs. As a result, Abbott-Bioline Malaria Ag *P.f*/*P.v* RDTs continued to be used widely, despite the growing concerns about their reliability. Finally in March 2025, the WHO-ISF team issued a notice concerning the use of “all malaria RDTs,” without specifically mentioning the Abbott-Bioline Malaria Ag *P.f*/*P.v* RDT. As far as we know there have not been major concerns about other WHO approved RDTs in this region. The WHO communication focused on the issue of “faint positive test lines,” and not the more critical concern; the high number of false-negative test results. As the WHO communication did not identify the specific brand that was underperforming, it cast doubt on all other RDTs in use. As a result, the WHO-ISF notice undermined confidence in malaria RDTs in general, even though these tests are essential for malaria control and elimination efforts in remote areas, where alternative diagnostic methods are unavailable.

**Fig. 7:**
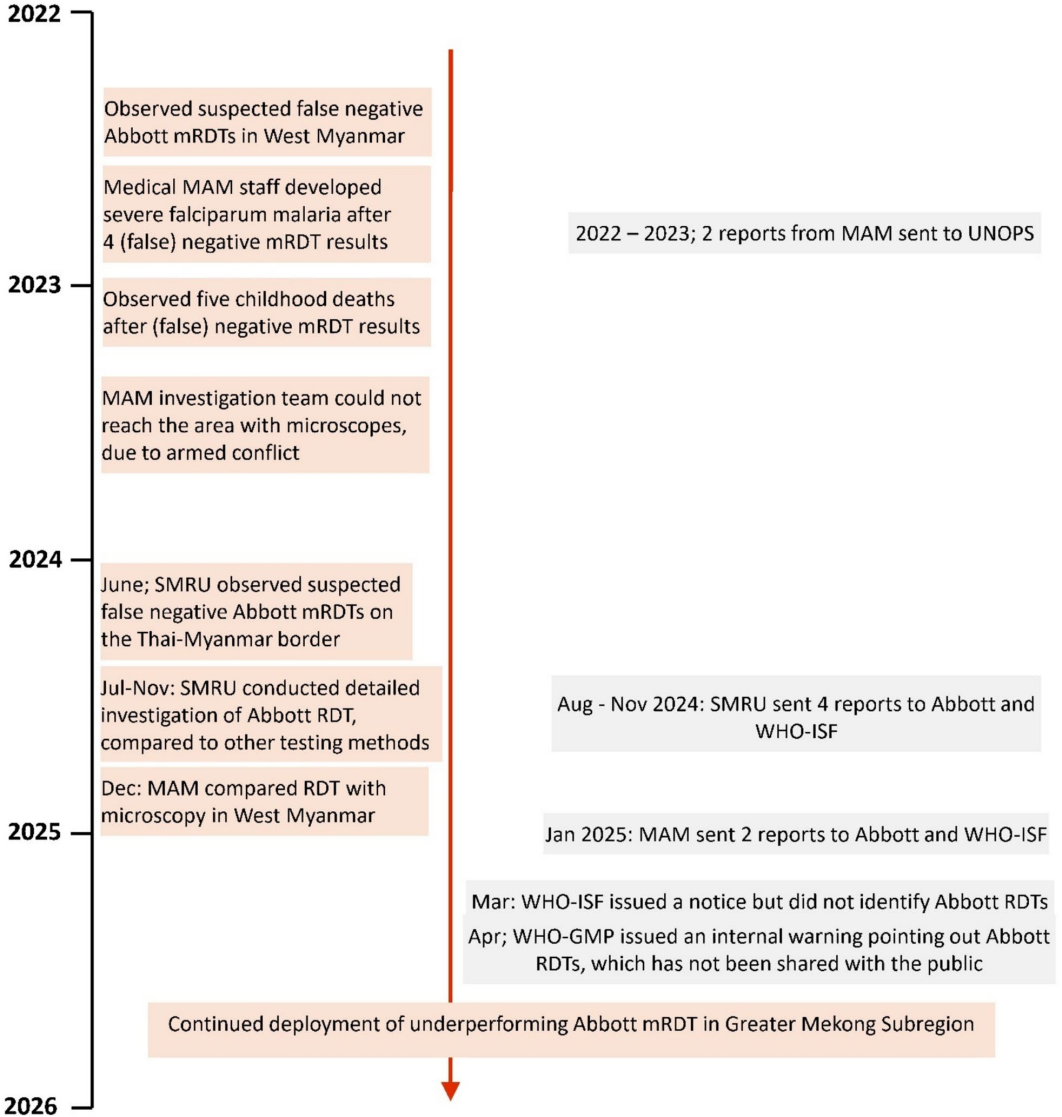
Timeline: the underperforming Abbott-Bioline Malaria Ag *P.f*/*P.v* RDT

The first clear warning came on the 30th of April 2025, in the form of a memo from WHO Global Malaria Programme (GMP), which reported problems with malaria RDTs manufactured by Abbott, mentioning that “Reports come from diverse field settings and experienced users, suggesting the issue is not related to user error or test interpretation” [11]. However, since this report was addressed only to the six regional WHO malaria supervisors, it is unclear whether most medical professionals in the GMS will be informed about the potential danger related to the use of Abbott-Bioline Malaria Ag *P.f*/*P.v* RDTs anytime soon. At the time of this writing (September 2025) organizations implementing malaria activities in the GMS with Abbott-Bioline Malaria Ag *P.f*/*P.v* RDTs are still not yet informed (Fig. 7).

This report has limitations. The survey did not assess *P. falciparum* malaria as the outbreak was with *P. vivax*. It evaluates only a very small proportion of all the Abbott-Bioline Malaria Ag *P.f*/*P.v* RDTs deployed in the region, although the results are consistent with reports from elsewhere.

There are many unresolved questions. We do not know the extent of the problem with these tests and we do not know what it is caused by, other than it appears to be a manufacturing rather than a storage or use issue. It also appears to be a new problem. Based on the studies elsewhere and our own experience the insensitivity appears to affect both the detection of *P. vivax* and *P. falciparum*. RDTs which produce bands which are undetectable or so faint that they can easily be missed are clearly not fit for purpose. The response to underperforming diagnostics should be managed in the same way that issues with low quality medicines are addressed.

## Conclusion

In this malaria survey conducted in Myanmar the Abbott-Bioline Malaria Ag *P.f*/*P.v* RDTs have proved to be insensitive, failing to detect a large proportion of vivax cases. This confirmed earlier suspicions and reports from elsewhere. Despite having been presented with clear evidence of poor test performance since August 2024, responses from both WHO-ISF and Abbott have been slow and unclear. This has undermined efforts to control and eliminate malaria and has resulted in preventable morbidity, and likely mortality.

## Supplementary Information

Additional file 1

## Data Availability

All relevant data are within the manuscript or supplementary information files. Upon reasonable request via the MORU website or from MORU data sharing committee datasharing@tropmedres.ac the raw data set will be available.

## Abbreviations

RDTs: Rapid diagnostic tests
*P.f*: *Plasmodium falciparum*
*P.v*: *Plasmodium vivax*
GMS: Greater Mekong Subregion
CHWs: Community health workers
HRP2: Histidine-rich protein 2
pLDH: Plasmodium lactate-dehydrogenase
WHO: The World Health Organization
UNOPS: United Nations Office for Project Services
MAM: Medical Action Myanmar
SMRU: Shoklo Malaria Research Unit
WHO-ISF: The World Health Organization Incidents and Substandard/Falsified Medical Products Team
NMCP: The National Malaria Control Programme
