# Supplementary material for: The underperforming Abbott-Bioline Malaria Ag P.f/P.v rapid diagnostic test: a whiter shade of pale - where the truth is not plain to see
Source: Malar J. 2025 Dec 15;24:441. doi: 10.1186/s12936-025-05638-6 (PMC12703916; doi:10.1186/s12936-025-05638-6)
Supplement: Supplementary file 1 — Additional file 1 [file 12936_2025_5638_MOESM1_ESM.docx]

**Supplementary material**

**Figure S1.** All 29 RDTs from patients who tested positive by microscopy with parasite counts ≥200/µL, together with their corresponding microscopy parasite count, RDT assessment, and ImageJ grey density line profile graphs.

**
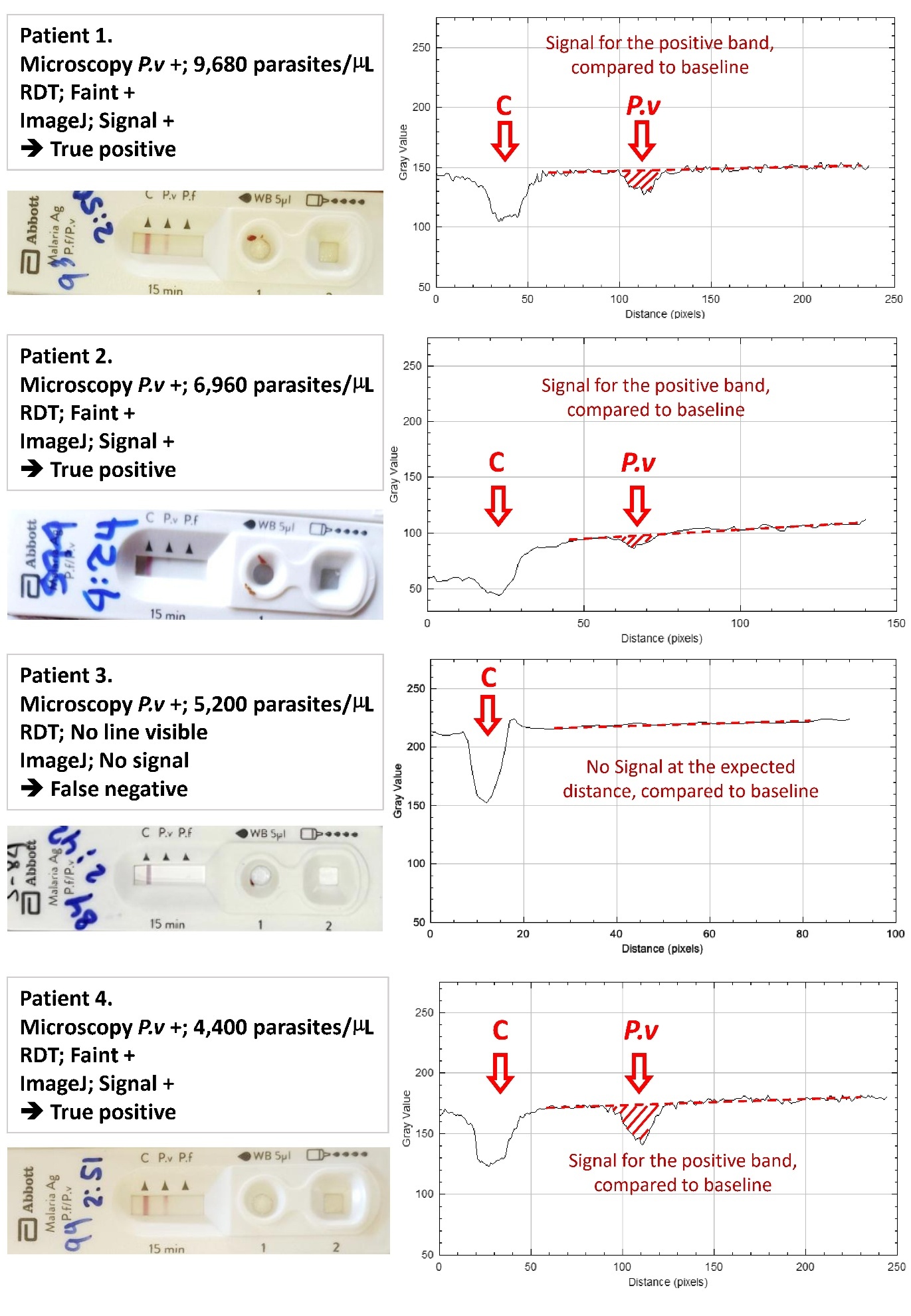
**

**
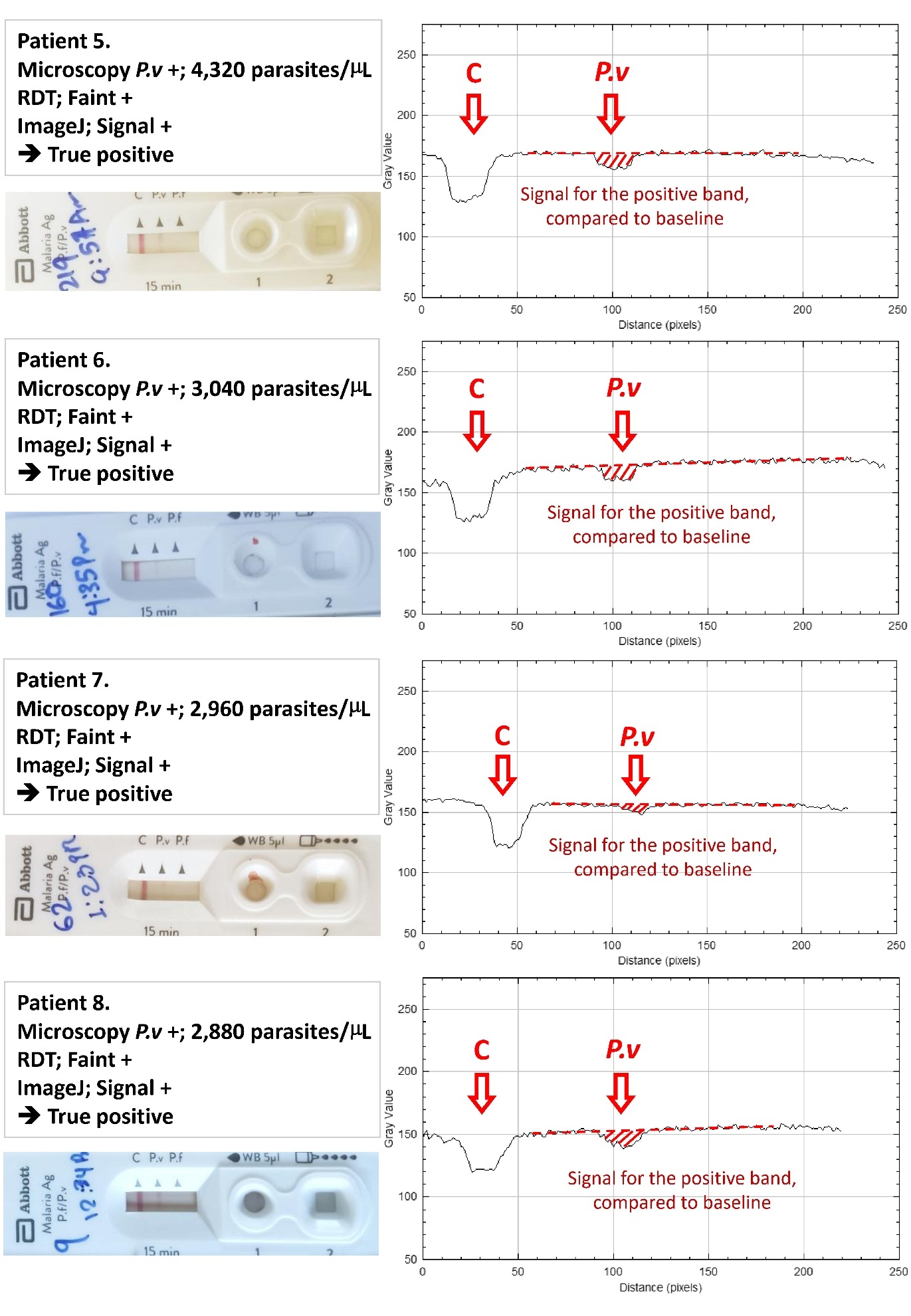
**

**
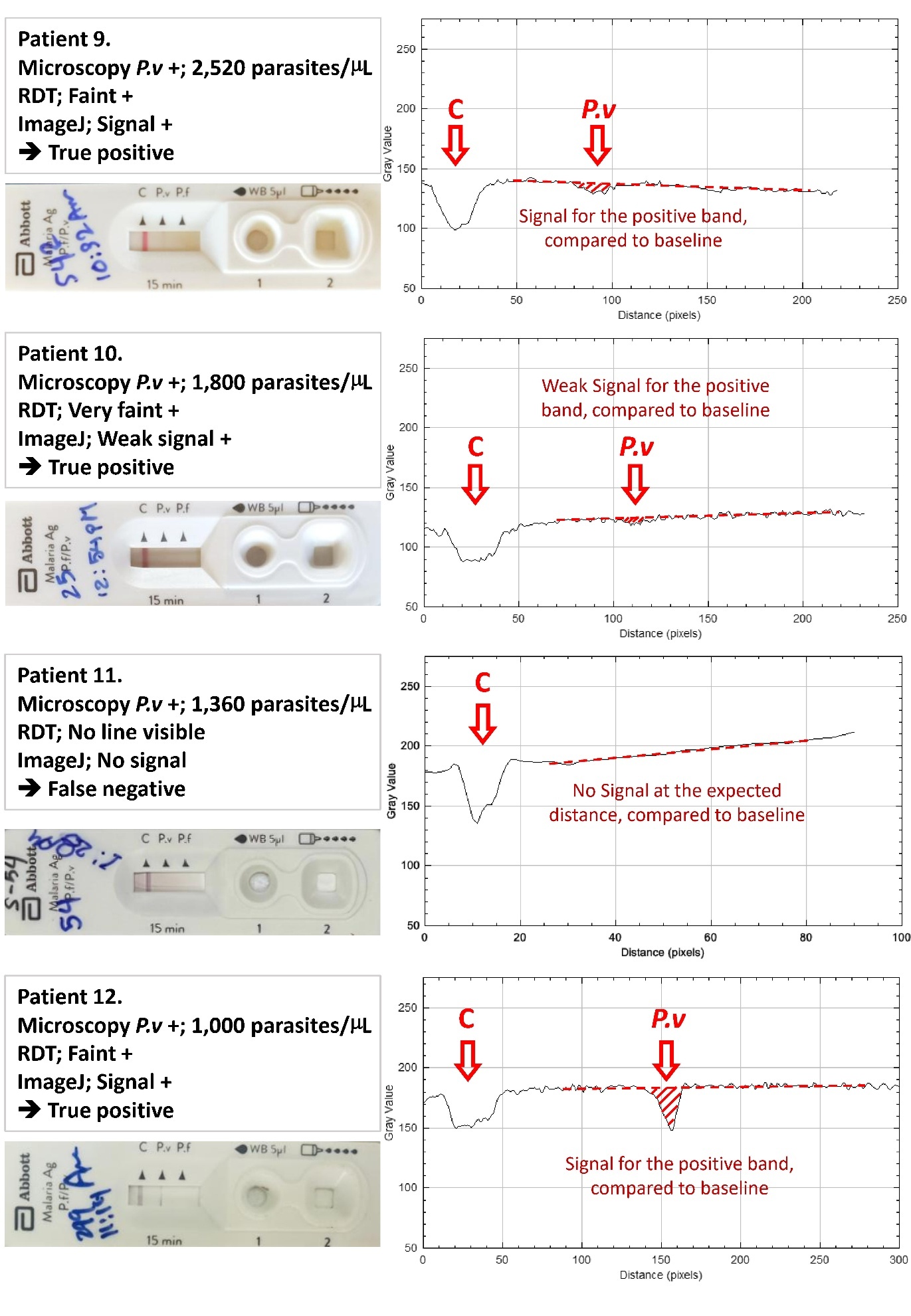
**

**
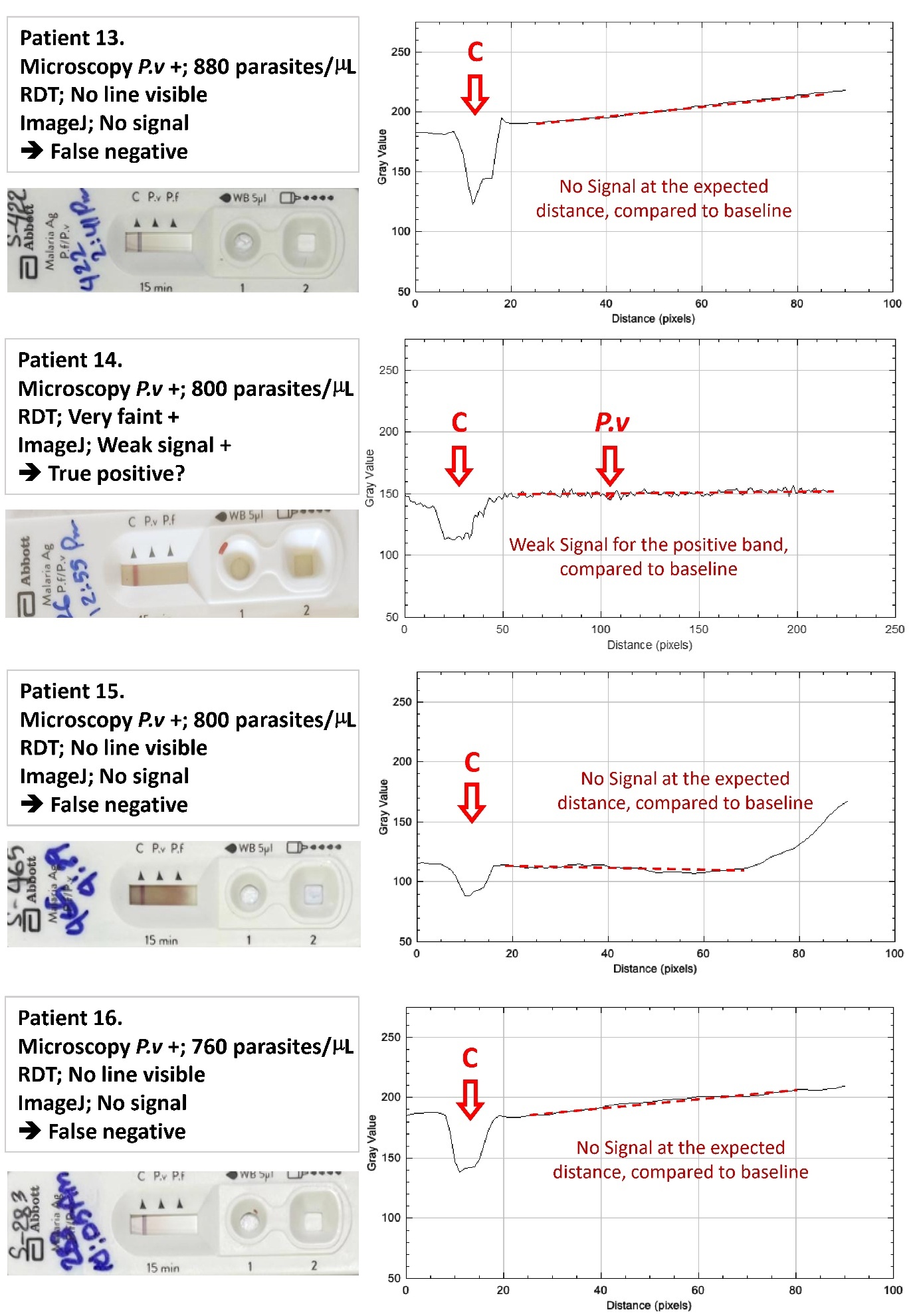
**

**
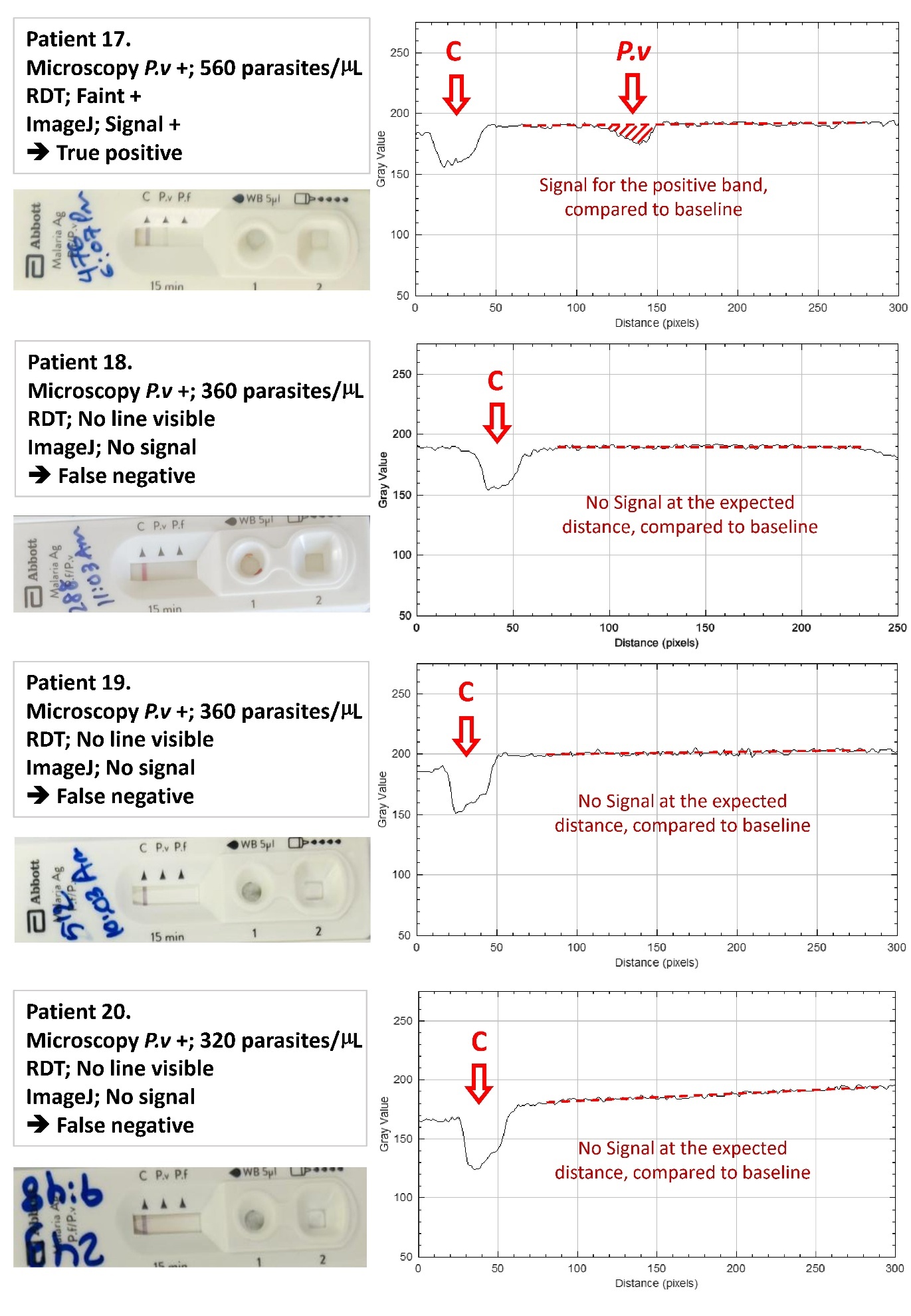
**

**
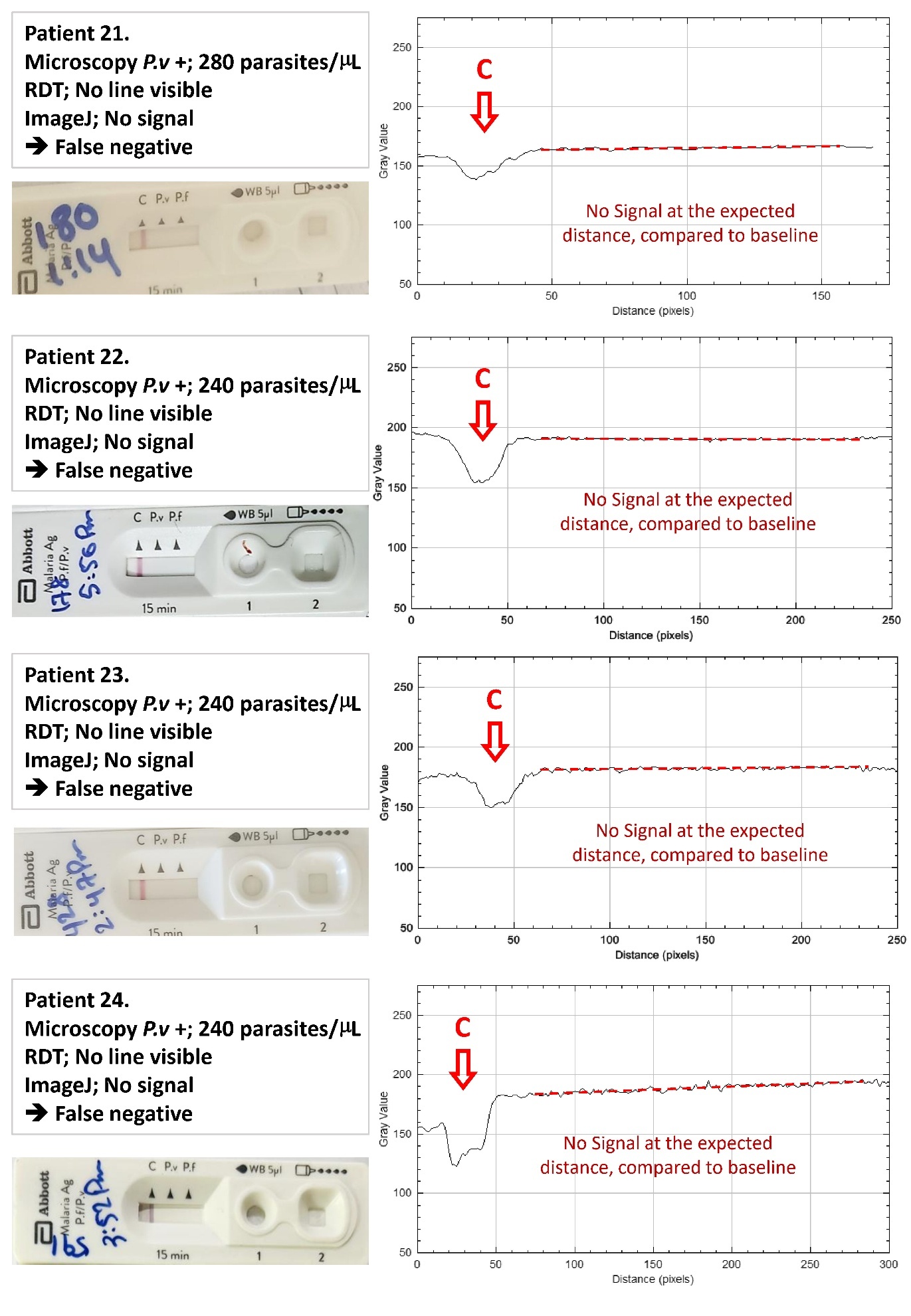
**

**
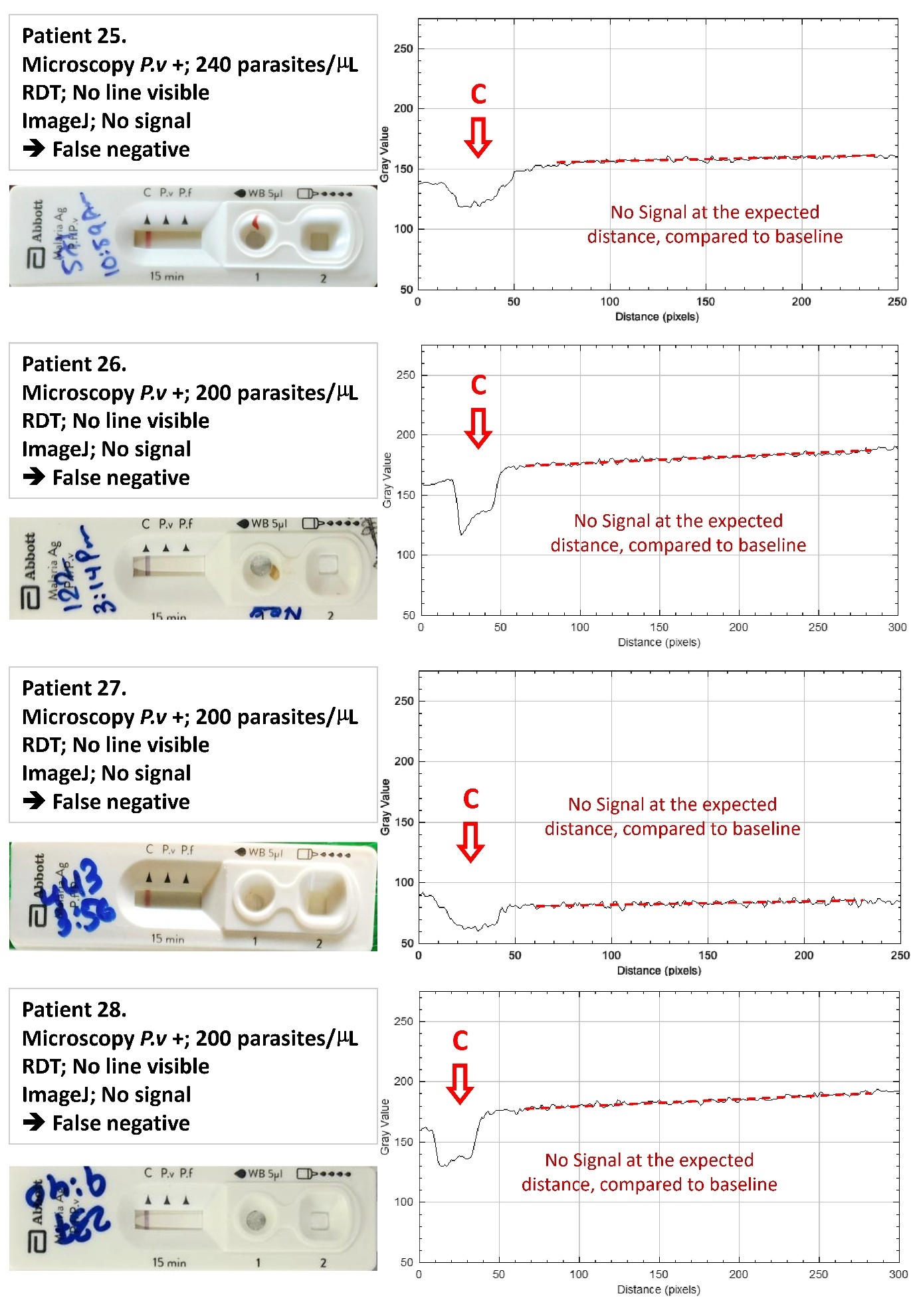
**

**
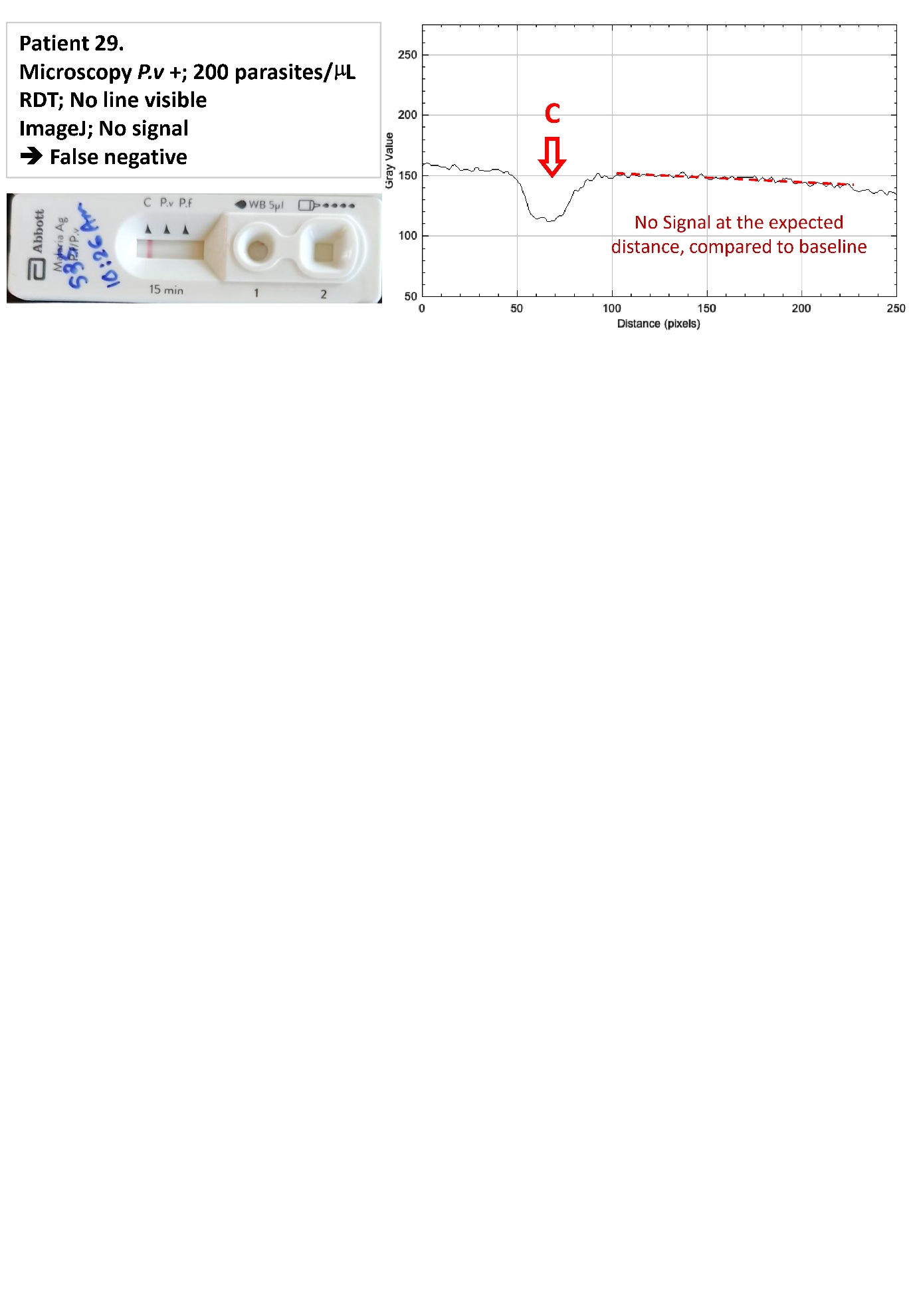
**
